# Dysregulation of the Norepinephrine Transporter Sustains Cortical Hypodopaminergia and Schizophrenia-Like Behaviors in Neuronal Rictor Null Mice

**DOI:** 10.1371/journal.pbio.1000393

**Published:** 2010-06-08

**Authors:** Michael A. Siuta, Sabrina D. Robertson, Heidi Kocalis, Christine Saunders, Paul J. Gresch, Vivek Khatri, Chiyo Shiota, J. Philip Kennedy, Craig W. Lindsley, Lynette C. Daws, Daniel B. Polley, Jeremy Veenstra-Vanderweele, Gregg D. Stanwood, Mark A. Magnuson, Kevin D. Niswender, Aurelio Galli

**Affiliations:** 1 Center for Molecular Neuroscience, Vanderbilt University School of Medicine, Nashville, Tennessee, United States of America; 2 Department of Molecular Physiology and Biophysics, Vanderbilt University School of Medicine, Nashville, Tennessee, United States of America; 3 Department of Pharmacology, Vanderbilt University School of Medicine, Nashville, Tennessee, United States of America; 4 Vanderbilt Kennedy Center for Research on Human Development, Vanderbilt University School of Medicine, Nashville, Tennessee, United States of America; 5 Department of Surgery, Children's Hospital of Pittsburgh of UPMC, and University of Pittsburgh School of Medicine, Pittsburgh, Pennsylvania, United States of America; 6 Department of Pharmacology, University of Texas Health Science Center, San Antonio, Texas, United States of America; 7 Department of Hearing and Speech Sciences, Vanderbilt University School of Medicine, Nashville, Tennessee, United States of America; 8 Department of Psychiatry, Vanderbilt University School of Medicine, Nashville, Tennessee, United States of America; 9 Tennessee Valley Healthcare System, Nashville, Tennessee, United States of America; 10 Department of Medicine, Vanderbilt University School of Medicine, Nashville, Tennessee, United States of America; Mount Sinai School of Medicine, United States of America

## Abstract

The mammalian target of rapamycin (mTOR) complex 2 (mTORC2) is a multimeric signaling unit that phosphorylates protein kinase B/Akt following hormonal and growth factor stimulation. Defective Akt phosphorylation at the mTORC2-catalyzed Ser473 site has been linked to schizophrenia. While human imaging and animal studies implicate a fundamental role for Akt signaling in prefrontal dopaminergic networks, the molecular mechanisms linking Akt phosphorylation to specific schizophrenia-related neurotransmission abnormalities have not yet been described. Importantly, current understanding of schizophrenia suggests that cortical decreases in DA neurotransmission and content, defined here as cortical hypodopaminergia, contribute to both the cognitive deficits and the negative symptoms characteristic of this disorder. We sought to identify a mechanism linking aberrant Akt signaling to these hallmarks of schizophrenia. We used conditional gene targeting in mice to eliminate the mTORC2 regulatory protein rictor in neurons, leading to impairments in neuronal Akt Ser473 phosphorylation. Rictor-null (KO) mice exhibit prepulse inhibition (PPI) deficits, a schizophrenia-associated behavior. In addition, they show reduced prefrontal dopamine (DA) content, elevated cortical norepinephrine (NE), unaltered cortical serotonin (5-HT), and enhanced expression of the NE transporter (NET). In the cortex, NET takes up both extracellular NE and DA. Thus, we propose that amplified NET function in rictor KO mice enhances accumulation of both NE and DA within the noradrenergic neuron. This phenomenon leads to conversion of DA to NE and ultimately supports both increased NE tissue content as well as a decrease in DA. In support of this hypothesis, NET blockade in rictor KO mice reversed cortical deficits in DA content and PPI, suggesting that dysregulation of DA homeostasis is driven by alteration in NET expression, which we show is ultimately influenced by Akt phosphorylation status. These data illuminate a molecular link, Akt regulation of NET, between the recognized association of Akt signaling deficits in schizophrenia with a specific mechanism for cortical hypodopaminergia and hypofunction. Additionally, our findings identify Akt as a novel modulator of monoamine homeostasis in the cortex.

## Abstract 2

A novel animal model highlights the link between Akt dysfunction, reduced cortical dopamine function, norepinephrine transporters, and schizophrenia-like behaviors.

## Author Summary

Schizophrenia is a disorder caused by multiple genetic and environmental variables. Despite the disease's heterogeneous causes, current hypotheses suggest that dysfunction of dopamine signaling in the brain is one of the final common pathways involved. One gene that may be involved encodes the protein kinase Akt, which is regulated by hormones, growth factors, and neurotransmitter receptors. In this study, we examined the potential molecular mechanisms linking Akt dysregulation to cortical hypodopaminergia, and ultimately to the pathology of schizophrenia. Using transgenic technology, we generated a mouse model with defective neuronal Akt signaling. Neurochemical and behavioral phenotypes associated with schizophrenia include decreases in prefrontal dopamine signaling and deficits in sensorimotor gating, two phenotypes we observed in our transgenic animals. Further, we observed that impaired cortical Akt activity significantly enhanced norepinephrine transporter function. Interestingly, we found that by blocking this transporter, we could reverse the cortical hypodopaminergia and behavioral deficits seen in our transgenic mice. The norepinephrine transporter is a presynaptic membrane protein that is critical for maintaining both norepinephrine and cortical dopamine homeostasis. Taken together, this work supports the potential for targeting both Akt and the norepinephrine transporter for treating dopamine-related mood disorders.

## Introduction

Mammalian target of rapamycin (mTOR) complex 2 (mTORC2) is one of two highly conserved protein kinases that are critical regulators of cell growth and metabolism. mTOR complex 1 (mTORC1) and mTORC2 are functionally distinct multiprotein complexes that are defined by their subunit composition, rapamycin sensitivity, and substrate selectivity. Raptor, mLST8, PRAS40, and mTOR comprise the rapamycin sensitive mTORC1 while the rapamycin insensitive mTORC2 contains rictor, mSIN1, mLST8, and mTOR. Two key substrates of mTORC1 are S6K and 4E-BP, which are important regulators of translation, while protein kinase B, also known as Akt, is the primary substrate of mTORC2 [1]. Specifically, mTORC2 is the kinase responsible for phosphorylation of Akt at serine residue 473, one of two key phosphorylation sites [1]. Akt is an extensively studied kinase that has been implicated in numerous disorders such as diabetes, obesity, cancer, and mental disorders such as schizophrenia [2]. Post-mortem, imaging, and genetic association studies in humans [3],[4],[5] reveal that Akt deficiencies are associated with schizophrenia. Genetic studies in rodents further corroborate the relationship between dysregulation in Akt signaling and disruptions in dopamine (DA)-associated behaviors linked to schizophrenia [4],[6].

Putative evidence for a role of defects in mTORC2 signaling in mental illnesses preceded the discovery of the mTORC2 complex itself. Indeed, lithium, used to treat bipolar disorder, stimulates phosphorylation of Akt at Ser473, the mTORC2 phosphorylation site [7]. The link between mTORC2 signaling deficits and mental illness has been strengthened by seminal work demonstrating that certain antidepressants [8], along with both typical [4] and atypical antipsychotics [9], increase Akt Ser473 phosphorylation. Furthermore, findings of diminished Ser473 phosphorylation and/or activity in post-mortem brains of patients with schizophrenia [10] and depression [11] potentially fortify the association between dysregulation of mTORC2-Akt signaling and development of psychiatric illnesses, although these findings may be confounded by perimortem artifacts [12]. Recent observations that blunted Ser473 phosphorylation occurs in lymphocytes derived from patients with schizophrenia and psychosis-prone normal individuals [13] also support the plausibility that mTORC2-Akt deficits are involved in schizophrenia.

While human imaging and animal studies implicate a fundamental role for Akt signaling in prefrontal DA networks, the molecular mechanisms linking mTORC2/Akt to schizophrenia-related neurotransmission abnormalities have been elusive [3],[6]. Importantly, models of schizophrenia suggest that cortical deficits in DA neurotransmission and content, defined here as cortical hypodopaminergia, contribute to both the cognitive deficits and the negative symptoms characteristic of this disorder [14]. Consistent with this hypothesis, imaging studies reveal that genetic variation associated with low activity Akt alleles interact epistatically with catechol-O-methyltransferase (COMT), a gene responsible for degradation of prefrontal synaptic DA. Together, these interactions ultimately affect the fidelity of prefrontal networks in humans [3] by decreasing DA availability at prefrontal synapses [15]. Thus, a compelling hypothesis in schizophrenia is that impaired mTORC2/Akt signaling triggers aberrant regulation of DA homeostasis.

Termination of DA signaling at prefrontal synapses involves two mechanisms: degradation via enzymes including COMT, and clearance via the norepinephrine (NE) transporter (NET) [16],[17],[18], which takes up both major brain catecholamines, DA and NE [17],[19]. Interestingly, insulin administration, which stimulates mTORC2/Akt signaling, decreases NET transcription in brain, while hypoinsulinemia and decreased mTORC2/Akt signaling increases NET transcription [20]. Therefore, we hypothesized that dysregulation of mTORC2/Akt signaling may provide a mechanistic link to cortical hypodopaminergia. Specifically, we propose that reduced Akt activity mediates increased NET expression and increased DA clearance by noradrenergic neurons in cortex, a novel molecular mechanism that explains how Akt dysfunction contributes to a reduction in prefrontal DA.

To test this hypothesis, we have generated an animal model in which mTORC2/Akt signaling down-regulation is achieved by neuronal deletion of a key mTORC2 regulatory subunit, rictor. We used a Cre-lox strategy to restrict the genetic deletion to neurons and bypass embryonic lethality associated with whole body deletion [21]. The goal of the present study is to test how alteration in Akt signaling affects DA homeostasis in the prefrontal cortex.

## Results

### Rictor Deletion Attenuates Akt Ser473 Phosphorylation

**Figure 1:**
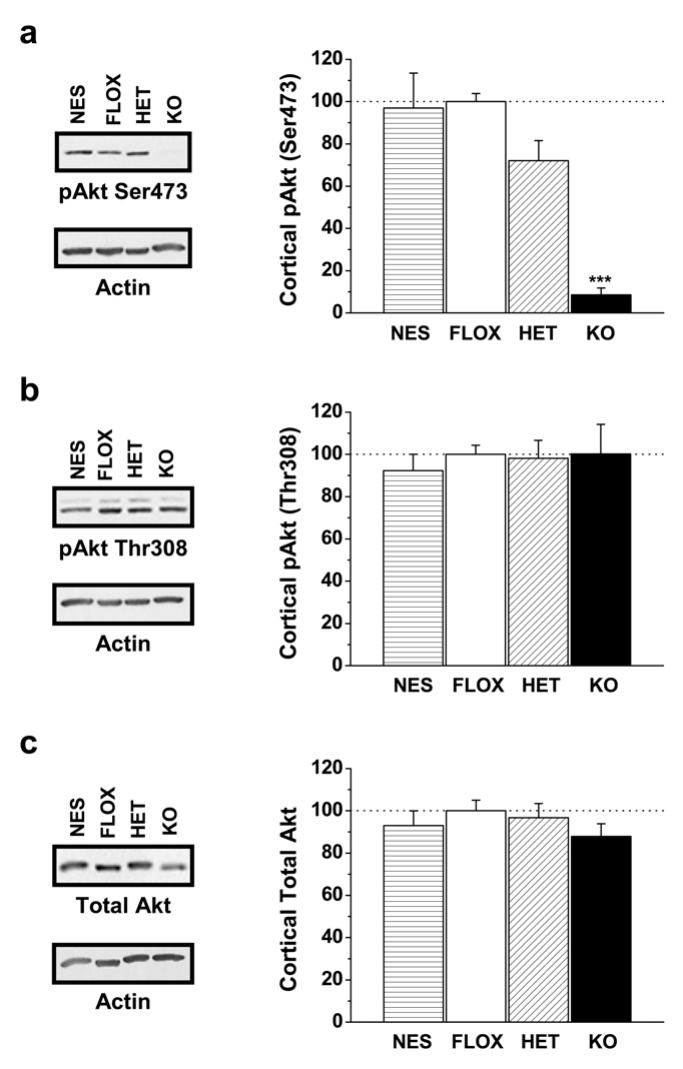
Neuronal rictor deletion specifically abolishes Akt phosphorylation at Ser473 in the cortex. (A) Phosphorylation of Akt on residue Ser473 in the cortex. (B) Phosphorylation of Akt at Thr308 and (C) total Akt in the cortex are similar for all genotypes. Shown are mean±s.e.m of optical densities as a percentage of FLOX control mice. Genotypes shown include animals expressing only nestin-CRE (NES), mice expressing two copies of the “floxed” rictor allele (FLOX) only, heterozygous mice which express nestin-CRE and a single copy of the “floxed” rictor allele (HET), and knockout mice expressing both nestin-CRE and two copies of the “floxed” rictor allele (KO). Total cortical protein extract was loaded in each lane. Representative immunoblots are shown, as probed with antibodies to phosphorylated Akt at Ser473 (A) Thr308 (B), total Akt (C), and actin to serve as a loading control. Samples *n* = 9–16. ***p<0.001 one-way ANOVA.

Akt deficiency is mechanistically linked to prefrontal cortex abnormalities and schizophrenia-linked phenotypes in several mouse models [6], although a clear molecular mechanism for how Akt regulates cortical function remains elusive. Here, we investigate the dopaminergic consequences of abolishing Akt phosphorylation at Ser473 in neurons by utilizing the Cre/LoxP system to delete rictor specifically in neurons. Mice were engineered with a floxed rictor allele, as previously described [21], and crossed with neuron-specific nestin gene (NES mice) Cre driver line. Validating our approach, rictor knockout (KO) mice lack rictor mRNA expression and rictor protein expression in a gene-dosage dependent manner within the brain and cortex (Figure S1; *p*<0.01 and *p*<0.05, respectively, by one-way ANOVA followed by Dunnett's test). Importantly for the current hypothesis, neuronal rictor deletion abolishes Akt phosphorylation at Ser473 within the cortex of rictor KO mice (Figure 1A; ***p<0.001 by one-way ANOVA Dunnett's test) compared to FLOX (floxed allele(s) in the absence of Cre), NES (Cre allele in the absence of a FLOX allele), and heterozygous rictor neuronal KO mice (HET). Phosphorylation of Akt at Thr308 (Figure 1B) and total levels of Akt (Figure 1C) within the cortex are not different among the genotypes, allowing a direct evaluation of the effects of Ser473 phosphorylation. Similarly, rictor deletion also abolishes Akt phosphorylation at Ser473 in other brain regions, such as the substantia nigra (SN)/ventral tegmental area (VTA) of rictor KO mice, while Thr308 phosphorylation and total levels of Akt are unaltered (data were normalized to control (FLOX) and reported as mean±s.e.m., *p* values by Student's *t* test; pAkt Ser473 FLOX = 100±13%, KO = 6±1%, *p*<0.001; pAkt Thr308 FLOX = 100±13%, KO = 89±8%, *p* = 0.49; total Akt FLOX = 100±13%, KO = 101±10%, *p* = 0.96). Furthermore, total protein levels of mTOR in the cortex are not altered by neuron-specific rictor KO (data were normalized to control (FLOX) and reported as mean±s.e.m.; FLOX = 100±9%, NES = 130±12%, HET = 118±12%, KO = 116±12%; *p* = 0.32 by one-way ANOVA followed by Dunnett's test).

### Neuronal Rictor KO Mice Display Sensorimotor Gating Deficits

**Figure 2:**
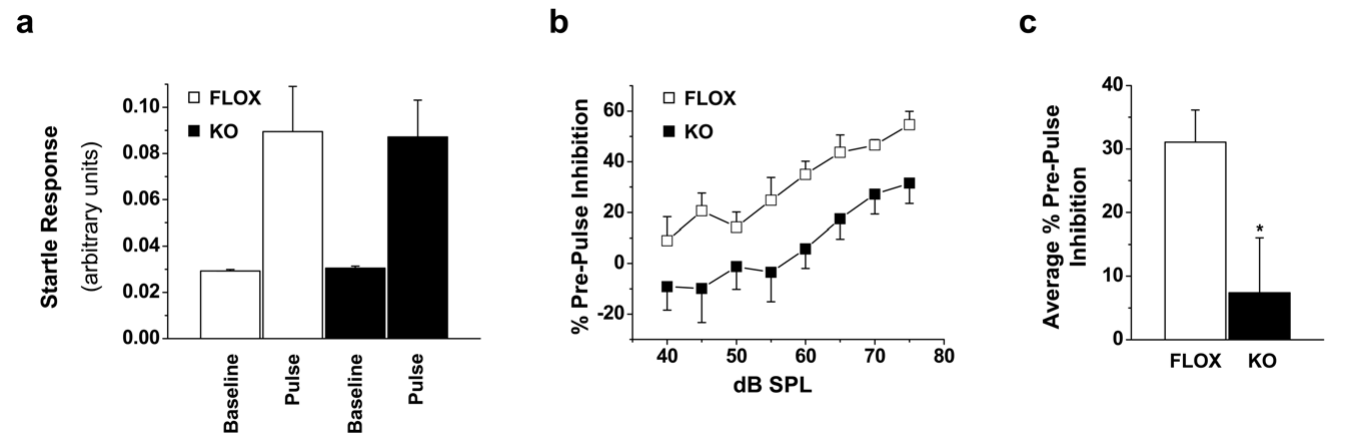
Neuronal rictor deletion results in sensorimotor gating deficits as assayed by PPI. (A) No difference in startle reflex elicited by a 94 decibel (dB) sound pressure level (SPL) noise was observed between rictor KO and littermate FLOX control mice. (B) The percentage of PPI was reduced significantly across the entire dB range in rictor KO mice. (C) The average PPI across the entire dB range reveals a significant deficit in PPI in rictor KO mice; *n* = 6–7 animals. *p<0.05 Student's *t* test.

Prepulse inhibition (PPI) behavior has long been identified as a promising phenotype for translational studies of schizophrenia owing to the direct parallels in expression between rodent and human subjects. While PPI deficits are present in psychiatric disorders other than schizophrenia, they have a clear heritable component in schizophrenic families and these deficits can be attenuated by antipsychotic drug administration. Furthermore, PPI deficits are also linked to the hypofunction of corticostriatal forebrain circuits that is characteristic of schizophrenia [22]. The PPI behavioral assay measures the degree to which the startle response elicited by a loud “pulse” sound is attenuated when immediately preceded by a non-startling “prepulse” sound. As such, it assays the degree to which a brief sensory trace can rapidly modify a subsequent motoric response, thereby representing a straightforward approach towards quantifying sensorimotor dysregulation, which is generally regarded as an endophenotype of schizophrenia. Since evidence suggests a role for Akt in schizophrenia, and rictor KO mice demonstrate profound deficits in Akt phosphorylation, we tested whether rictor KO mice display impaired PPI relative to FLOX control mice. No differences in startle responses elicited by a 94 dB sound pressure level (SPL) noise burst were observed, suggesting that hearing and gross motor function were similar between groups (Figure 2A; Student's *t* test; *p* = 0.44). By contrast, analysis of PPI behavior revealed clear differences between genotypes; startle reflex amplitude was inhibited at all prepulse intensities in FLOX control mice, with the amount of PPI increasing monotonically from 40 to 75 dB SPL (Figure 2B). In rictor KO mice, prepulse sound levels between 40 and 55 dB did not appreciably attenuate the startle reflex. PPI was not observed in rictor KO mice until prepulse levels >55 dB, albeit at a weaker level in comparison to FLOX mice. These differences gave rise to a significant reduction of PPI across prepulse sound levels (Figure 2B; *p<0.05 by ANOVA). This PPI deficit can also be expressed as a significant decrease in average PPI across all sound levels in rictor KO mice (Figure 2C; Student's *t* test, *p<0.05). Thus neuronal rictor deletion, with loss of Akt Ser473 phosphorylation, impairs the forebrain circuits critically involved with sensorimotor integration. Given the strong evidence for PPI deficits in schizophrenic patients, we hypothesize that this mouse model has the potential to lend novel insight into the molecular mechanisms by which Akt deficits contribute to the schizophrenic phenotype.

### Rictor KO Mice Display Hypodopaminergia in Rostral Cortex

**Figure 3:**
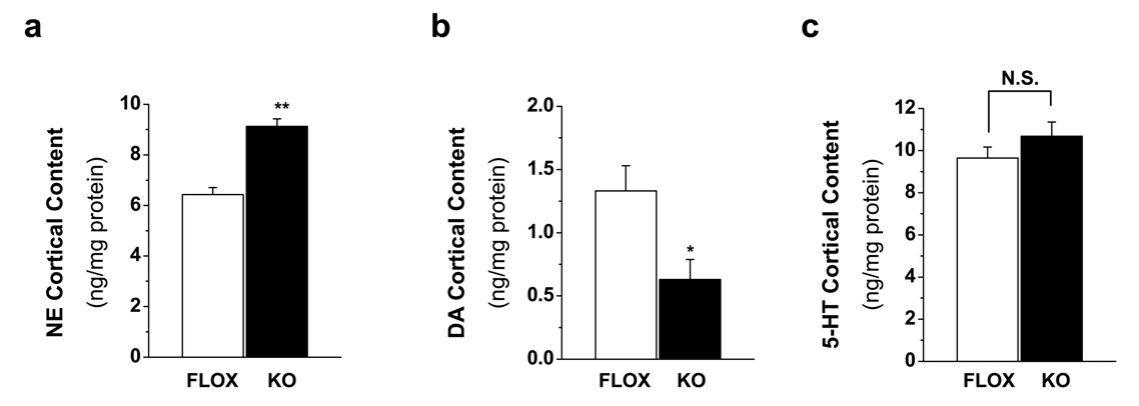
Monoamine content in the rostral cortex is significantly altered in rictor KO mice. Tissue content of (A) NE, (B) DA, and (C) serotonin (5-HT) in rostral cortical homogenates. Results are presented as mean±s.e.m ng/mg of protein, *n* = 4–10. *p<0.05; **p<0.01 Student's *t* test.

For almost 50 years, schizophrenia research has centered on dopaminergic signaling as a crucial component of the etiology of the disease [23],[24],[25]. In particular, the original “DA hypothesis” heavily supported the notion of excessive DA neurotransmission and DA content, defined here as hyperdopaminergia, within the brain [26],[27]. Subsequent revision of the hypothesis, however, has transformed thinking from a global hyperdopaminergia to a regional hyperdopaminergia within the striatum and dopaminergic hypofunction within the cortex [14],[28],[29]. While this conceptualization is an oversimplification of a highly complex disorder, we utilized this hypothesis to hone in on molecular mechanisms that contribute to cortical hypodopaminergia. Furthermore, previous studies have linked pre-frontal DA deficits with PPI deficits in animal models, and perhaps the PPI deficits observed in the rictor KO mice could be partially explained by alterations in cortical DA content [30],[31]. Thus, we investigated steady state levels of DA, serotonin (5-HT), and NE in a region of mouse brain that is roughly analogous to the human prefrontal cortex and contains areas such as the intralimbic, prelimbic, and anterior cingulate cortex. Interestingly, HPLC with electrochemical detection of DA, NE, and 5-HT in the PFC of rictor KO mice revealed striking alterations in the DA and NE tissue content of these animals, while 5-HT levels remained unchanged (Figure 3). While both DA and NE levels are significantly different in rictor KO mice, they change in opposite directions; NE tissue content is significantly increased (Figure 3A; **p<0.01 by one-way ANOVA followed by Dunnett's test) while DA levels are significantly decreased (Figure 3B; Student's *t* test, *p<0.05). In addition, NES mice show similar PFC DA content levels (6.4±0.2 ng/mg protein) as FLOX mice. Thus, rictor KO mice display a key feature of the “dopamine hypothesis” of schizophrenia, namely hypodopaminergia in the rostral cortex, which may explain the sensorimotor gating deficits described earlier. Importantly, 5-HT levels are unaltered in the cortex (Figure 3C; Student's *t* test, *p* = 0.26) indicating that rictor deletion does not simply result in global monoaminergic alterations but rather specific changes in the dopaminergic and noradrenergic systems. In addition, extracellular levels of DA were determined in the PFC of rictor KO mice by microdialysis. Under basal conditions, extracellular DA is not significantly different in rictor KO mice compared to FLOX controls (data are reported as pg of DA/µL, mean±s.e.m.; FLOX = 0.54±0.15, KO = 0.76±0.18, *n* = 4–5; *p* = 0.35 by Student's *t* test). While basal extracellular levels of DA are unaltered in rictor KO mice, these animals do display significant deficits in DA tissue content, suggesting that maintenance of DA homeostasis is perturbed.

While prefrontal hypodopaminergia has been linked to PPI deficits, other studies clearly demonstrate a link between striatal hyperdopaminergia and PPI deficits [32],[33]. Thus, we sought to determine if rictor deletion increases tissue levels of DA in the striatum or in projecting DA neurons from the SN and VTA. DA levels in the SN/VTA are not altered in rictor KO mice (data are reported as ng of DA/mg protein, mean±s.e.m.; FLOX = 4.4±0.5, KO = 4.8±0.4, *p* = 0.60 by Student's *t* test). Importantly, similar to the cortex, DA tissue content in the striatum of rictor KO mice is significantly decreased (data are reported as ng of DA/mg protein, mean±s.e.m.; FLOX = 101.2±7.7, KO = 80.1±3.4, *p<0.05 by Student's *t* test). Thus, our data indicate that the PPI deficits observed in rictor KO mice are likely to arise from impairments in DA neurotransmission.

### Rictor Deletion Increases NET Expression and Function

It is intriguing that DA content is decreased while NE content is increased in rictor KO mice. Importantly, decreases in mTORC2/Akt signaling induced by hypoinsulinemia have been shown to increase NET transcription [20]. Moreover, early studies and unpublished data from our laboratory implicate deficits in Akt signaling with not only increases in NET transcription but also acute increases in NET cell surface expression (i.e. intact Akt signaling decreases NET availability at the plasma membrane) [20],[34],[35],[36]. Thus, we predict that altered DA homeostasis in rictor KO mice is due to changes in NET cell surface expression mediated by impaired Akt phosphorylation. Importantly, unlike other brain regions where DAT is the primary mechanism for removing DA from the synapse, in cortex DAT contributes relatively little and NET performs the majority of DA clearance. Indeed NET has a higher affinity for DA than NE itself, but DA can also be degraded in the synapse by COMT [16],[17]. Given the pivotal role of rictor in Akt regulation and the role of Akt signaling in determining NET availability, we hypothesize that rictor KO mice will display aberrant NET regulation that sustains the alterations in NE and DA levels seen in the rostral cortex.

**Figure 4:**
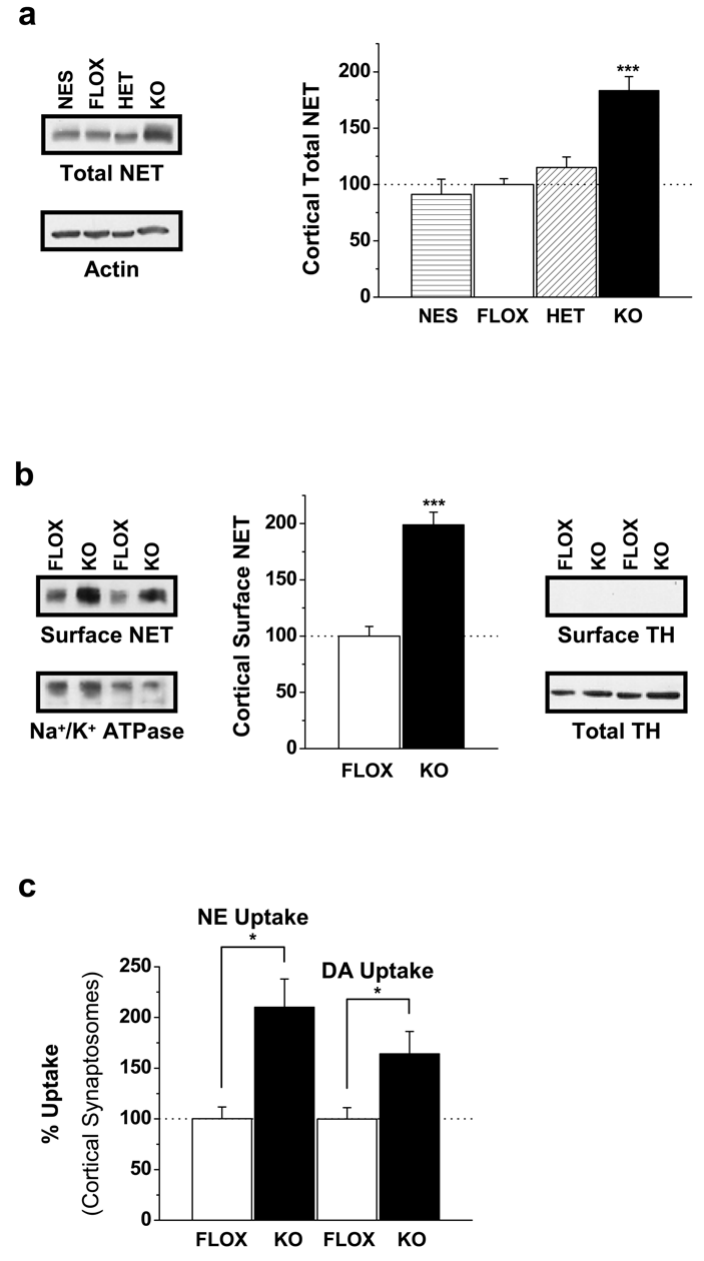
Neuronal rictor deletion results in increased NET expression and function. (A) NET protein levels in the cortex. Mean±s.e.m optical densities are shown as a percentage of FLOX control mice. Representative immunoblots are shown, as probed with antibodies to NET, and actin (loading control); *n* = 10. (B) Levels of surface NET as measured from the biotinylated fraction of cortical slices. Mean±s.e.m optical density is shown as a percentage of FLOX control mice. Representative immunoblots are shown, as probed with antibodies to NET, Na^+^/K^+^ ATPase to serve as plasma membrane/loading control (*n* = 3–5), and TH which is absent in the biotinylated fractions since it is a cytosolic protein. (C) [^3^H]NE and [^3^H]DA uptake into cortical synaptosomes of FLOX and KO mice. Mean±s.e.m uptake is shown as a percentage of uptake in FLOX control mice; *n* = 12–18. *p<0.05; ***p<0.001 Student's *t* test.

As hypothesized, total cortical NET protein is increased approximately 2-fold in rictor KO mice compared to all other genotypes (Figure 4A; ***p<0.0001 by one-way ANOVA followed by Dunnett's test). Furthermore, biotinylation assays reveal that cell surface levels of NET are also significantly increased (Figure 4B; Student's *t* test, ***p<0.0001). Tyrosine hydroxylase (TH), a cytosolic protein, was detected exclusively in the total protein fraction but not in the surface fraction, indicating that the biotinylated fraction represents exclusively cell surface proteins. Finally, the striking enhancement in surface NET detected in rictor KO mice results in a significant increase in NET function as assayed by cortical synaptosomal NE uptake (Figure 4C; Student's *t* test, *p<0.05). The nearly 2-fold increase in cortical synaptosomal NE uptake was also observed for DA (Figure 4C; Student's *t* test, *p<0.05) indicating that rictor deletion increases DA clearance by NET in noradrenergic neurons and as a consequence reduces cortical DA content. Furthermore, DA content is not decreased due to increased degradation since COMT levels were not different in cortex compared to FLOX control mice (data were normalized to control (FLOX) and reported as mean±s.e.m.; FLOX = 100±8%, NES = 78±11%, HET = 130±24%, KO = 80±11%; *p* = 0.10 by one-way ANOVA followed by Dunnett's test). Thus, the increase in NET expression and function within the cortex of rictor KO mice has the potential to mechanistically explain both the increased NE tissue content and decreased cortical DA tissue content described earlier (Figure 3). Interestingly, we did not find a significant difference in serotonin transporter expression within the PFC of the rictor KO mice, as measured by citalopram binding (unpublished data).

While our data indicate that global neuronal mTORC2 dysfunction enhances NET function and induces cortical hypodopaminergia, we sought to demonstrate more specifically that these alterations could arise from downregulation of cortical Akt activity. Thus, we utilized the isoform specific Akt1 inhibitor [37],[38],[39] in cortical slices to show that Akt inhibition is capable of directly determining NET surface availability (Figure S2). Surface levels of NET are significantly enhanced in biotinylated cortical slices treated with the Akt1 inhibitor (Figure S2A; *p<0.05 by Student's *t* test). Importantly, the levels of Akt Ser473 phosphorylation are substantially diminished in samples of these inhibitor treated slices (Figure S2B; ***p<0.0001). Together, these data support the notion that Akt stimulated regulation of the transporter occurs not only at the level of transcription, as is seen in rictor KO mice, but also at the level of transporter trafficking. Furthermore, the ability of Akt inhibition to enhance NET surface expression in cortical slices indicates that all the molecular machinery necessary for this rictor/Akt regulation of NET is intact within the PFC and thus is consistent with our hypothesis that altered cortical monoamine homeostasis via aberrant NET regulation underlies PPI deficits in rictor KO mice.

We hypothesize that amplified NET function in rictor KO mice enhances the accumulation of both NE and DA within the noradrenergic neuron leading to conversion of DA to NE and ultimately supporting both increased NE tissue content and a state of hypodopaminergia. Such a mechanism within the prefrontal cortex provides an elegant molecular mechanism linking Akt hypophosphorylation to both cortical hypodopaminergia and PPI deficits, two key hallmarks of schizophrenia.

### Midbrain Dopaminergic Neurons and Cortical Monoaminergic Projections Are Unaltered in Rictor KO Mice

**Figure 5:**
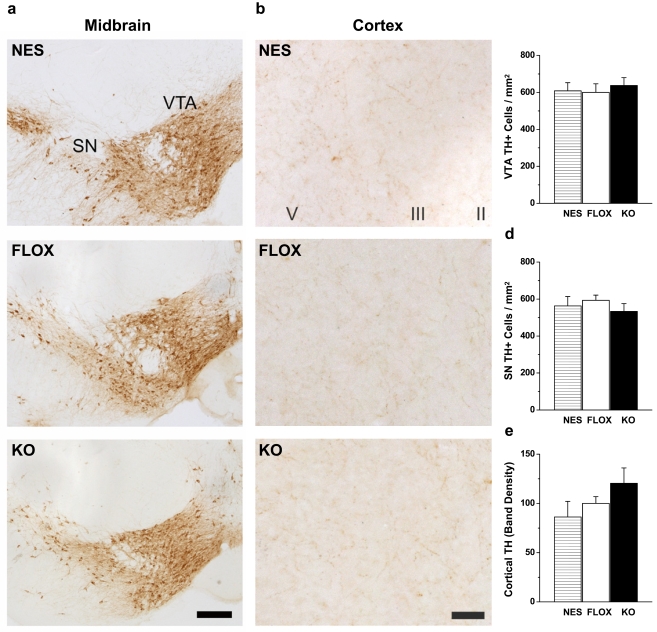
TH staining and expression in the midbrain and cortex is similar in NES, FLOX, and KO mice. TH immunoreactivity in the (A) midbrain substantia nigra (SN) and ventral tegmental area (VTA) and the (B) cortex. Scale bars = 50 µm. Coronal brain sections were stained with TH antibody and cell counts of TH^+^ cells were taken. Cell counts are similar in NES, FLOX, and KO matched mice in both the (C) VTA and the (D) SN. Mean±s.e.m TH^+^ cells/mm^2^ are shown; *n* = 6. (E) TH protein levels in the cortex. Mean±s.e.m optical densities are shown as a percentage of FLOX control mice; *n* = 19–22. One-way ANOVA analysis reveals no significant difference between genotypes.

Considering the widespread function of Akt and its role in cell growth and proliferation, we next sought to demonstrate that the changes in DA and NE levels within the cortex were specifically due to increased NET expression rather than global changes in the number or projections of dopaminergic and noradrenergic neurons. While rictor KO mice do display a gross reduction in brain size, similar to what is seen for Akt3 deficient mice (brain weight normalized to body weight; FLOX 2.49±0.18% compared to KO 1.63±0.10%; *p*<0.0005 by one-way ANOVA followed by Dunnett's test), coronal brain sections stained for TH revealed no significant alterations in dopaminergic cell number within the VTA or SN (Figure 5A and 5C–5D; VTA *p* = 0.82 by one-way ANOVA, SN *p* = 0.53 by one-way ANOVA). Furthermore, TH staining of dopaminergic and noradrenergic projections within the cortex do not reveal any gross alterations among the groups (Figure 5B). The immunostaining was confirmed with Western blot analysis of total cortical TH protein levels (Figure 5E; *p* = 0.20 by one-way ANOVA). Other markers of dopaminergic neurons in the cortex were not significantly altered in the rictor KO mice such as total levels of D2 DA receptors (data were normalized to control (FLOX) and reported as mean±s.e.m.; FLOX = 100±5%, NES = 95±13%, HET = 96±9%, KO = 108±18%; *p* = 0.85 by one-way ANOVA followed by Dunnett's test). These data demonstrate that the DA and NE systems in the rictor KO mice are not globally altered. Therefore, we propose that the changes in cortical NE and DA tissue content can be primarily accounted for by the specific enhancement of NET expression in noradrenergic neurons.

### NET Blockade Reverses the Sensorimotor Gating Deficits and Hypodopaminergia in Rictor KO Mice

**Figure 6:**
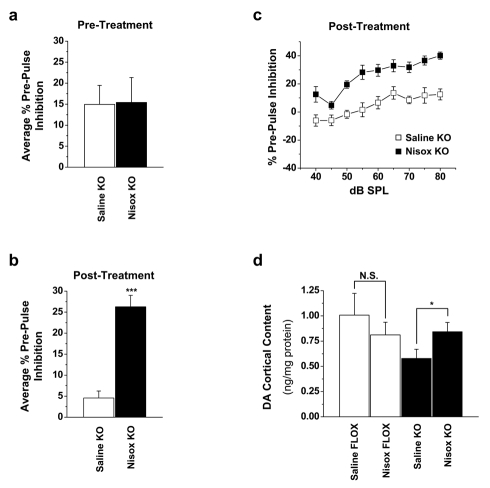
Nisoxetine restores PPI deficits and DA levels in the rostral cortex of rictor KO mice. (A) No differences are observed in the average %PPI across the experimental range of dB in KO mice prior to treatment with saline or nisoxetine. (B) Average %PPI 30 min after i.p. injection of either saline or nisoxetine (30 mg/kg) in KO mice reveals a significant increase in %PPI in nisoxetine treated KO mice. (C) The mean PPI across all sound levels in KO mice treated with either saline or nisoxetine for 30 min; *n* = 5–6, ***p<0.001 Student's *t* test. (D) 8 d of nisoxetine treatment restores DA levels in the rostral cortex of rictor KO mice; *n* = 4–6. *p<0.05 Student's *t* test.

We hypothesize that aberrant Akt phosphorylation in rictor KO mice results in enhancement of NET function within the cortex, resulting in changes in both DA and NE homeostasis. If this model is valid, then NET inhibition should reverse both PPI behavioral deficits and cortical deficits in DA tissue content. In order to test this hypothesis, we treated rictor KO mice with nisoxetine (NET specific blocker) or saline. Prior to treatment, both groups of rictor KO mice demonstrated comparable startle reflex amplitude similar to Figure 2B (unpublished data). The average PPI across all sound levels also were not different between the two rictor KO groups prior to treatment (Figure 6A; Student's *t* test, *p* = 0.95). Following the initial PPI trial, the rictor KO mice received i.p. injections of either saline or nisoxetine (30 mg/kg) and 30 min later began a second PPI trial. Nisoxetine reversed rictor KO PPI deficits compared to saline treated animals with a ∼5-fold increase of average PPI (Figure 6B Student's *t* test, ***p<0.0001). Similar to FLOX control mice (Figure 2B), nisoxetine treated rictor KO mice display startle reflex amplitudes that are inhibited by all prepulse intensities, with the amount of PPI increasing monotonically from 40–75 dB SPL (Figure 6C). Importantly, saline treated animals exhibited PPI at prepulse levels>55 dB, albeit at a weaker level as compared to nisoxetine treated animals, consistent with Figure 2B. Thus, nisoxetine treatment significantly reverses the PPI deficits observed in rictor KO mice (Figure 6C; *p*<0.0001 by two-way ANOVA) providing support for our model of mechanistic linkage between Akt, NET, and cortical DA and NE homeostasis.

In addition to nisoxetine treatment, we sought to determine if traditional antipsychotics were capable of reversing the PPI deficits displayed in rictor KO mice. Unlike nisoxetine, acute clozapine treatment (i.p. 3 mg/kg for 30 min prior to PPI) did not rescue PPI deficits in rictor KO mice (data are reported as average percent PPI mean±s.e.m. as in Figure 2C; saline-KO 24.3±8.6%, clozapine-KO 20.5±6.4%; Student's *t* test, *p* = 0.73). Interestingly, previous studies have shown that antipsychotic treatment enhances activity and phosphorylation of Akt at Ser473, and this increase is hypothesized to be important for the efficacy of such drugs [4],[40],[41]. Consistently, FLOX mice subjected to the same clozapine treatment as described above show significantly enhanced cortical phosphorylation of Akt at Ser473 (data are normalized to total levels of Akt and expressed as percent of control mean±s.e.m.; saline-FLOX 100±20%, clozapine-FLOX 215±50%; *p<0.05 by one-way ANOVA). Importantly, the same treatment does not alter Ser473 phosphorylation of Akt in rictor KO mice (data are normalized to total levels of Akt and expressed as percent of control (saline treated FLOX) mean±s.e.m.; saline-KO 5±1%, clozapine-KO 5±2%; *p*>0.05 by one-way ANOVA). Thus, the inability of clozapine to rescue PPI deficits in rictor KO mice may be partially due to the genetic neuronal deletion of rictor, which abolishes the ability of clozapine to enhance Akt Ser473 phosphorylation in these animals. These data are consistent with the hypothesis that Akt deficits play an important role in the etiology of schizophrenia and that perhaps some degree of antipsychotic efficacy is due to their ability to enhance Akt function.

The success of nisoxetine treatment in rescuing the PPI deficits in rictor KO mice was consistent with our model and led us next to determine if NET inhibition could normalize prefrontal cortex DA tissue content. In this experiment, rictor KO and FLOX control mice received either nisoxetine (30 mg/kg) or saline injections i.p. daily for 8 consecutive days and were euthanized 30 min following their last i.p. injection. DA levels were measured in the rostral cortex by electrochemical detection. Similar to the results seen in Figure 3, saline treated rictor KO mice had reduced DA tissue content levels in comparison to saline treated controls (Figure 6D; Student's *t* test, *p<0.04) and 8 d of nisoxetine treatment significantly enhanced DA content (Figure 6D; Student's *t* test, *p<0.03) while the same treatment in FLOX control mice did not have a significant impact on DA levels (Figure 6D; Student's *t* test, *p* = 0.21). Moreover, chronic nisoxetine treatment does not significantly alter cortical NE tissue content in either FLOX control or rictor KO mice (data are reported as ng of NE/mg protein, mean±s.e.m.; FLOX-saline = 5.7±0.4, FLOX-nisoxetine = 6.5±0.5, Student's *t* test, *p* = 0.29; KO-saline = 8.3±0.7, KO-nisoxetine = 8.6±0.4, Student's *t* test, *p* = 0.74). These data further support the model that enhanced NET expression alters DA content in the rictor KO mice and indicate that hypodopaminergia (and PPI deficits) can be rectified with NET specific inhibition *via* nisoxetine. The observations that chronic nisoxetine administration rescues DA content and acute treatment reverses impaired PPI are consistent with a model whereby impairment of PPI is due to decreased intra-synaptic DA in rictor KO mice. Our data suggest that reduced intra-synaptic DA content could arise from enhanced clearance of DA via NET.

NET specific inhibition with nisoxetine has been utilized to rectify PPI deficits in some animal models of schizophrenia [42]. Only a small number of studies, however, have investigated the utility of NET specific inhibition in schizophrenic patients with a particular focus on symptoms related to cortical hypofunction, and the efficacy of such treatment is still controversial [43]. Currently, a number of additional clinical trials with substantial sample sizes are ongoing and promise to reveal if NET specific inhibition is therapeutic in schizophrenia, especially for cognitive symptoms such as poor concentration and memory (clinicaltrials.gov).

## Discussion

The “DA hypothesis” for schizophrenia has enjoyed a resurgence of interest, and increasing evidence links Akt to DA related behaviors, yet key molecular mechanisms linking Akt to DA homeostasis have been elusive. Given evidence for a role of Akt in the regulation of NET expression and function in cortex and evidence that NET transports DA in this brain area, it has been our priority to develop a compelling experimental model to uncover a molecular link between Akt and cortical DA homeostasis. Here, we conducted the first studies, to our knowledge, in an animal model where a genetic deletion that disrupts Akt phosphorylation enhances expression of NET and leads to a cortical hypodopaminergic phenotype with schizophrenia-linked behavioral consequences.

Our data are consistent with pathophysiological models of schizophrenia that emphasize “hypofrontality” of DA systems. Given the role of NET in DA clearance in prefrontal synapses [16],[17],[44], Akt-linked changes in NET expression may thereby translate to cognitive deficits and negative symptoms [14]. These data, as well as our proof-of-principle results in mice, lead to the compelling hypothesis that NET inhibition would have therapeutic potential to selectively enhance DA tone in the prefrontal cortex and perhaps alleviate negative symptoms. Consistent with this reasoning, several clinical trials are currently investigating NET blockers for cognitive deficits in patients with schizophrenia.

Our data demonstrate that neuronal mTORC2 dysfunction is sufficient to generate cortical hypodopaminergia and schizophrenia-linked behaviors. In particular, we show that genetic mTORC2 disruption impairs PPI, a schizophrenia-linked phenotype, which has been validated in genetic mouse models of the disorder [22]. An emerging body of evidence associates Akt phosphorylation deficits with mental illness in humans, giving our findings of impaired Akt phosphorylation in mice more translational viability [4],[13]. For example, studies show diminished Akt1 protein content in lymphocytes of patients with schizophrenia [4],[13]. In concert with our findings, candidate gene approaches aimed at identifying genetic variation in proteins associated with mTORC2/Akt signaling pathways, including rictor and other mTORC2 subunit proteins like mSin1 [45], may yield new insights into the genetic basis of mental illness.

While DA is classically implicated in the pathogenesis of schizophrenia, concepts of neurotransmitter dysfunction in this disease process are constantly evolving. This is reflected by popular glutamatergic and recently proposed revisions to dopaminergic hypotheses of schizophrenia [29]. However, the role of elevated cortical NE, while not typically emphasized, should not be overlooked. Indeed, findings of elevated NE in CSF of patients with schizophrenia, which led to noradrenergic hypotheses of schizophrenia in the early 1980s [46], support our model by which increased cortical NET expression leads to increased NE content but decreased DA content. While the sensitivity of these measurements to acute stressors made the reproducibility and reliability of these methods in the aforementioned studies questionable, a role for elevated NE in the development of schizophrenia-like phenotypes in humans cannot be completely ruled out. Indeed, our current findings, as well as others, intimately and mechanistically link DA alterations together with NE changes [47]. As a recent revision to the DA hypothesis of schizophrenia suggests, schizophrenia could be conceptualized as a disease where DA dysfunction is a “final common pathway” that can be elicited by a number of more proximal causes, including both genetic and epigenetic factors and disruption in other neurotransmitter systems [29], and including, as we propose here, increased NET function.

Schizophrenia is thought to arise from rather complex gene-environment interactions, and therefore, acquired (rather than monogenetic) dysfunction in mTORC2/Akt signaling is a particularly intriguing mechanism. For example, acquired Akt defects are associated with impaired regulation of blood glucose and diabetes, which is overrepresented in first episode, medication-naive patients with schizophrenia [48]. mTORC2/Akt signaling also provides a promising portal into “multiple hit” models of schizophrenia, as Akt is positioned to interact with other candidate genes such as neuregulin-1 and COMT [3],[49] as well as environmental risk factors for schizophrenia including obstetric complications and early life stressors [8],[50]. The effects of neuronal mTORC2 dysfunction on NET expression observed in our study ultimately illustrates a potential molecular mechanism to link disparate genetic and environmental factors (i.e. obesity/diabetes/insulin resistance) to dysfunction in a putative “final common pathway” of schizophrenia, namely alterations in DA signaling [29]. Indeed, while more remains to be learned about mTORC2, deficiencies in Akt signaling even in “healthy” individuals are associated with impaired prefrontal cortex activation on working memory tasks [3] and proneness to psychosis, suggesting a subtle influence on brain function and behavior that may require other genetic and environmental hits to result in clinical disease. Thus, our data provide one molecular mechanism, NET regulation, towards a framework linking environmental stressors and/or lifestyle factors to mTORC2/Akt signaling and ultimately to DA-dependent behaviors. Our studies provide a potential molecular mechanism linking Akt dysfunction to a schizophrenia-like phenotype and suggest the viability of targeting both Akt phosphorylation and NET as pharmacotherapies for schizophrenia.

## Methods

All procedures were performed according to Vanderbilt University Institutional Animal Care and Use Committee approved procedures.

### Generation of Mice

All mice were fully backcrossed to C57Bl6 background. Mice homozygous for an allele containing LoxP sites flanking exon 3 of the rictor gene (rictor f/f Nes−/−; FLOX) were crossed with neuron specific Nestin cre transgenic mice (rictorw/w Nes+/+; NES) obtained from Jackson Laboratories to create double heterozygous (rictorf/w NesCre+/−; HET) offspring (w is the wildtype allele). HET mice were then crossed to produce neuron specific rictor KO mice (rictor f/f Nes+/+ or +/−; KO) and HET KO mice. Control animals were of the following genotypes (rictorf/f Nes−/− FLOX, rictorf/w Nes−/− and rictorw/w Nes+/+, Nes+/− or Nes−/−). Subsequent crossings between FLOX and HET mice were used to generate additional study animals. Genotyping was performed by PCR using DNA obtained from tail clippings with primers for the floxed, nestin, and recombined alleles as described [21].

### mRNA Expression

Total brain RNA was extracted from Trizol reagent (Invitrogen). cDNA was synthesized with a High Capacity cDNA reverse transcription kit (Applied Biosystems). rictor mRNA was quantified with real time RT PCR on a Bio-Rad iCycler using iQ SYBR green Supermix reagent (Bio-Rad) and primer pairs as reported in [21]. Expression was normalized to levels of the housekeeping gene RPL13.

### Immunoblot Analysis

Mice were anesthetized with volatile isoflurane after which rapid decapitation allowed brain removal. Brains were chilled on ice and dissected using a scope for either total cortex, rostral cortex, or caudal cortex. Tissue was homogenized and lysed in a Triton based buffer that contained a cocktail of protease inhibitors plus NaF (2 mM) and sodium orthovanadate (2 mM). After homogenization samples were centrifuged at 17,000× g for 30 min, and the supernatant was collected and processed for protein concentration determination. For Western blotting, ≈30 µg of protein per sample was run on a 10%–12% acrylamide gel, transferred to a PVDF membrane blocked with 5% milk, and incubated with primary antibodies to a variety of proteins. Akt, rictor, and mTOR antibodies were obtained from Cell Signaling. We also used actin (Sigma), Na+/K+ ATPase (DBH), NET (Mab Technologies Inc. NET05-2), and TH (Chemicon/Millipore) antibodies. Secondary antibodies were obtained from Santa Cruz Biotechnology. After chemiluminescent visualization on Hyper-film ECL film, protein band densities were quantified and analyzed (Scion Image; http://www.scioncorp.com).

### PPI

Startle responses were elicited with a 94 dB SPL broadband noise burst (2–75 kHz, 50 ms duration, 0 ms rise/fall time) and measured through a floor plate mounted on piezo force transducers (PCB Piezotronics). In two-thirds of the trials, the noise burst (pulse) was preceded by a prepulse (2.4–78.2 kHz frequency-modulated sweep, 40 octaves/s, 0.14 ms stimulus onset asynchrony) ranging from 40–75 dB SPL. Startle responses were measured across 297 trials (20±6.4 s intertrial interval, initial 27 trials discarded to minimize within-session habituation), as max–min of the force signal within 400 ms following onset of the noise burst. Presence of a normal startle response (significant difference between force plate amplitude −600 to −200 ms (baseline) versus 0–400 ms (startle) from noise burst onset) was a prerequisite for subsequent analysis to minimize possible contributions of gross sensorimotor deficits in either genotype. This criterion excluded 2/8 WT and 0/7 rictor KO mice from further analysis. Inhibition of the startle reflex was quantified for each prepulse intensity as PPI = 100×((pulse-alone)−(prepulse+pulse)/pulse alone).

### Tissue Extraction for Neurochemistry

Brain sections were homogenized in 100–750 ul of 0.1 M TCA, which contains 10-2 M sodium acetate, 10-4 M EDTA, 5 ng/ml isoproterenol (as internal standard), and 10.5% methanol (pH 3.8). Samples were spun in a microcentrifuge at 10,000 g for 20 min, the supernatant removed and stored at −80°C. The pellet was saved for protein analysis. Supernatant was then thawed and spun for 20 min and then analyzed for biogenic monoamines and/or amino acids by a specific HPLC assay (Vanderbilt Neurochemistry Core). Biogenic amines were eluted in the following order: NE, MHPG, epinephrine, DOPAC, DA, 5-HIAA, HVA, 5-HT, and 3-MT (2).

### In Vivo Microdialysis

Mice were anesthetized with isoflurane and placed in a stereotaxic frame using mouse-specific ear bars (Kopf Instruments, Tujunga, CA). A guide cannula (CMA7 microdialysis, USA) was placed above the medial prefrontal cortex (+2.0 AP, ±0.7 ML from Bregma and −1.0 DV from skull for FLOX or NES mice and +1.9 AP, ±0.6 ML from Bregma and −1.0 DV from skull for rictor KO mice) and secured to the skull with epoxy adhesive (Plastics one). Animals were allowed to recover from the surgery (1–3 d). The day before the experiment, animals were placed in individual dialysis chambers and the microdialysis probe (CMA7 microdialysis, USA) with the active length of 2 mm was inserted into the guide cannula. One end of a tether (Plastics One) is attached to a harness and the other end attached to a swivel (Instech) that is mounted on a counterbalanced arm above the dialysis chamber. The probe was perfused overnight at a flow rate of 0.5 µL/min with artificial cerebral spinal fluid containing 149 mM NaCl, 2.8 mM KCl, 1.2 mM CaCl2, 1.2 mM MgCl2, 5.4 mM d-glucose, pH 7.2. On the day of the experiment the flow rate was changed to 1.0 µL/min and after equilibration dialysis fractions (20 min each) were collected to establish baseline concentrations of neurotransmitter efflux. Dialysate samples were stored at −80°C and analyzed by HPLC-EC for DA levels. Probe placement was verified after collection of slices by Nissl staining of coronal slices.

### Brain Slice Preparation and Biotinylation

Brain slices were prepared from 6- to 10-wk-old mice that were anesthetized with isoflurane and rapidly decapitated. Following brain removal, the brain was chilled in oxygenated ≈4°C sucrose solution (sucrose 210 mM; NaCl 20 mM; KCl 2.5 mM; MgCl2 1 mM; NaH2PO4•H2O 1.2 mM), and then while in sucrose solution 300 µm coronal slices were made using a vibratome. Slices were then collected in oxygenated artificial cerebral spinal fluid (ACSF) (NaCl 125 mM, KCl 2.5 mM, NaH2PO4•H2O 1.2 mM, MgCl2 1 mM, CaCl2•2H2O 2 mM). For in vitro drug treatments slices were then allowed to recover for 1 h at 37°C in oxygenated ACSF (NaCl 125 mM, KCl 2.5 mM, 1 nM insulin, NaH2PO4•H2O 1.2 mM, MgCl2 1 mM, CaCl2•2H2O 2 mM) with either vehicle or Akt1 inhibitor and following recovery slices were then biotinylated. For non-treated slices, slices were immediately washed twice with oxygenated 4°C ACSF following collection, and then incubated with 4°C ACSF solution containing 1 mg/mL of EZ-Link Sulfo-NHS-SS-Biotin (Pierce/ThermoScientific; Rockford, IL) for 45 min. Slices were then washed twice with oxygenated 4°C ACSF, and then incubated with 4°C ACSF solution containing 1 mg/mL of EZ-Link Sulfo-NHS-SS-Biotin (Pierce Chemical; Rockford, IL) for 45 min. After biotin incubation, the slices were rinsed twice quickly and for two 10 min washes in oxygenated 4°C ACSF. The reaction was quenched by washing twice for 20 min each with oxygenated 4°C ACSF containing glycine. Following quenching, slices were frozen on dry ice and the cortex was cut out and frozen at −80°C until used. Slices were lysed in 1% Triton buffer (25 mM Hepes, 150 mM NaCl, 2 mM sodium orthovanadate, 2 mM NaF, plus a cocktail of protease inhibitors) and centrifuged at 17,000 g for 30 min at 4°C. After isolation of supernatant 0.1% Triton pulldown buffer (25 mM HEPES, 150 mM NaCl, 2 mM sodium orthovanadate, 2 mM NaF, plus a cocktail of protease inhibitors) was added. Protein concentration was determined using Bio-Rad's protein concentration kit. Biotinylated proteins were then isolated using ImmunoPure immobilized streptavidin beads (Pierce) overnight at 4°C with agitation. Beads were washed three times with 0.1% Triton pulldown buffer and biotinylated proteins were then eluted in 50 µL of 2× SDS-PAGE sample loading buffer at 95°C and then cooled to room temperature. Total slice lysates and the biotinylated (slice surface) fraction underwent immunodetection for NET (MabTechnoligies Inc., Stone Mountain, GA) as described previously.

### Synaptosomal Uptake

Mice were sacrificed by rapid decapitation and cortex dissected using an ice cold metal block and then homogenized at 400 rpm in at least 10 volumes (w/v) of ice-cold 0.32 M glucose with a Teflon-glass homogenizer (Wheaton Science Products, Millville, NJ). After centrifugation of the homogenate at 800 g for 10 min at 4°C, the supernatant was again centrifuged at 10,000 g for 15 min. The final pellet was gently resuspended in Krebs-Ringer HEPES (KRH) medium containing 118 mM NaCl, 4.8 mM KCl, 25 mM NaHCO3, 1 mM NaH2PO4, 1.3 mM CaCl2, 1.4 mM MgSO4, 10 mM glucose, 1 mM tropolone, 0.1 mM pargyline, and 0.1 mM ascorbic acid, pH 7.4, and protein concentration assayed. Aliquots (0.2 ml) of synaptosomal preparations (50 ng of protein) were prepared on ice. DA and NE transport assays (5 min at 37°C) were initiated by the addition of [3H]DA or [3H]NE (∼100 Ci/mmol, Amersham) and were terminated by immediate filtration over 0.3% polyethylenimine-coated glass fiber filters using a cell harvester (Brandel Inc., Gaithersburg, MD). The filters were washed three times with 1.5 ml of ice-cold phosphate-buffered saline (PBS) and incubated overnight in Ecoscint H (National Diagnostics, Atlanta, GA). Radioactivity bound to filters was counted using a Beckman LS 6,000 liquid scintillation counter. Nonspecific uptake, defined as the accumulation of [3H]DA or [3H]NE in the presence of 1 µM desipramine, was subtracted from total uptake values to obtain specific uptake values.

### TH Immunohistochemistry

Mice (*n* = 6/genotype) were anesthetized and transcardially perfused with 4% paraformaldehyde. Brains were removed and cryopreserved, and coronal sections were cut on a microtome at 40 µm and stained using minor modifications of published protocols. Sections were blocked in 4% Blotto (Nestlé Carnation dried milk), 0.2% Triton-X 100 in PBS, and incubated at 4°C for 3 d with a monoclonal anti-TH antibody (Sigma, 1∶4,000). Sections were thoroughly washed and then incubated in biotinylated anti-mouse IgG (Jackson Immunoresearch, 1∶1,000) for 60 min. Avidin-biotin amplification (Vectastain ABC Standard, Vector Labs) and 3-3′-diaminobenzidine reactions were used to visualize labeled proteins. Sections from all genotypes were processed in parallel to minimize variability between groups. Negative controls in which primary antibody was omitted revealed no specific labeling. Slides were coded so that the investigator was blinded to the genotype and sections were imaged using a Zeiss Axioskop microscope and Axiocam HR. TH-immunoreactive (IR) cells were counted in images obtained with a 40× objective in two fields per hemisphere derived from two to three sections per animal. Values were corrected for cell diameter, but no differences in cell diameter were found across genotypes.

### Statistical Analysis

All data are expressed as the mean±s.e.m. Statistical significance between groups was determined using *t* tests or one- and two-way ANOVAs followed by post hoc tests when the main effect or interaction was significant at *p*<0.05. Statistical analyses were conducted using software from Graph-Pad Prism. The number of animals and specific statistical analyses used in each experiment are indicated in the results and figure legend sections.

## Supporting Information

Figure S1 **Rictor mRNA levels and protein expression in the brain are reduced in a gene-dosage dependent manner.** (A) qRT-PCR confirms down-regulation of the rictor gene in HET and KO mice. Mean±s.e.m relative expression shown as a percentage of FLOX control mice; *n* = 4–5 animals. (B) Rictor protein levels in the cerebral cortex. Mean±s.e.m optical densities are shown as a percentage of FLOX control mice; *n* = 5, *p<0.05; **p<0.01 one-way ANOVA.
(0.40 MB TIF)

Figure S2 **Akt1 inhibition enhances NET surface availability in cortical slices.** (A) Levels of NET as measured from the different fractions (surface, total) of cortical slices from NES control mice. Slices were treated with either vehicle-DMSO (CTR) or 12 µM of the Akt1 inhibitor. Mean±s.e.m optical density were normalized to total NET and are shown as a percentage of CTR. Representative immunoblots are shown, as probed with antibodies to NET, Na^+^/K^+^ ATPase to serve as plasma membrane/loading control; *n* = 4, *p<0.05 Student's *t* test. (B) Phosphorylation of Akt on residue Ser473 measured from the same samples as (A). Mean±s.e.m of optical densities normalized to total Akt and shown as a percentage of CTR; *n* = 4, ***p<0.001 Student's *t* test.
(0.67 MB TIF)

## Abbreviations

COMT: catechol-O-methyltransferase
DA: dopamine
KO: knockout
mTOR: mammalian target of rapamycin
mTORC2: mTOR complex 2
mTORC1: mTOR complex 1
NE: norepinephrine
NET: norepinephrine transporter
PPI: prepulse inhibition
SN: substantia nigra
SPL: sound pressure level
TH: tyrosine hydroxylase
VTA: ventral tegmental area

## References

[1] Sarbassov D. D, Guertin D. A, Ali S. M, Sabatini D. M (2005). Phosphorylation and regulation of Akt/PKB by the rictor-mTOR complex.. Science.

[2] Beaulieu J. M, Gainetdinov R. R, Caron M. G (2009). Akt/GSK3 signaling in the action of psychotropic drugs.. Annu Rev Pharmacol Toxicol.

[3] Tan H. Y, Nicodemus K. K, Chen Q, Li Z, Brooke J. K (2008). Genetic variation in AKT1 is linked to dopamine-associated prefrontal cortical structure and function in humans.. J Clin Invest.

[4] Emamian E. S, Hall D, Birnbaum M. J, Karayiorgou M, Gogos J. A (2004). Convergent evidence for impaired AKT1-GSK3beta signaling in schizophrenia.. Nat Genet.

[5] Dawn L. T, Vladimir I. V, Po-Hsiu K, Joseph M, Brandon W (2008). AKT1 is associated with schizophrenia across multiple symptom dimensions in the Irish study of high density schizophrenia families.. Biological Psychiatry.

[6] Lai W. S, Xu B, Westphal K. G, Paterlini M, Olivier B (2006). Akt1 deficiency affects neuronal morphology and predisposes to abnormalities in prefrontal cortex functioning.. Proc Natl Acad Sci U S A.

[7] Chalecka-Franaszek E, Chuang D. M (1999). Lithium activates the serine/threonine kinase Akt-1 and suppresses glutamate-induced inhibition of Akt-1 activity in neurons.. Proc Natl Acad Sci U S A.

[8] Krishnan V, Han M-H, Mazei-Robison M, Iñiguez S. D, Ables J. L (2008). AKT signaling within the ventral tegmental area regulates cellular and behavioral responses to stressful stimuli.. Biological Psychiatry.

[9] Lu X. H, Dwyer D. S (2005). Second-generation antipsychotic drugs, olanzapine, quetiapine, and clozapine enhance neurite outgrowth in PC12 cells via PI3K/AKT, ERK, and pertussis toxin-sensitive pathways.. J Mol Neurosci.

[10] Zhao Z, Ksiezak-Reding H, Riggio S, Haroutunian V, Pasinetti G. M (2006). Insulin receptor deficits in schizophrenia and in cellular and animal models of insulin receptor dysfunction.. Schizophr Res.

[11] Karege F, Perroud N, Burkhardt S, Schwald M, Ballmann E (2007). Alteration in kinase activity but not in protein levels of protein kinase B and glycogen synthase kinase-3[beta] in ventral prefrontal cortex of depressed suicide victims.. Biological Psychiatry.

[12] Ide M, Ohnishi T, Murayama M, Matsumoto I, Yamada K (2006). Failure to support a genetic contribution of AKT1 polymorphisms and altered AKT signaling in schizophrenia.. J Neurochem.

[13] Keri S, Seres I, Kelemen O, Benedek G (2009). The relationship among neuregulin 1-stimulated phosphorylation of AKT, psychosis proneness, and habituation of arousal in nonclinical individuals.. Schizophr Bull.

[14] Davis K. L, Kahn R. S, Ko G, Davidson M (1991). Dopamine in schizophrenia: a review and reconceptualization.. Am J Psychiatry.

[15] Lotta T, Vidgren J, Tilgmann C, Ulmanen I, Melen K (2002). Kinetics of human soluble and membrane-bound catechol o-methyltransferase: a revised mechanism and description of the thermolabile variant of the enzyme.. Biochemistry.

[16] Miner L. H, Schroeter S, Blakely R. D, Sesack S. R (2003). Ultrastructural localization of the norepinephrine transporter in superficial and deep layers of the rat prelimbic prefrontal cortex and its spatial relationship to probable dopamine terminals.. J Comp Neurol.

[17] Moron J. A, Brockington A, Wise R. A, Rocha B. A, Hope B. T (2002). Dopamine uptake through the norepinephrine transporter in brain regions with low levels of the dopamine transporter: evidence from knock-out mouse lines.. J Neurosci.

[18] Gresch P. J, Sved A. F, Zigmond M. J, Finlay J. M (1995). Local influence of endogenous norepinephrine on extracellular dopamine in rat medial prefrontal cortex.. J Neurochem.

[19] Yamamoto B. K, Novotney S (1998). Regulation of extracellular dopamine by the norepinephrine transporter.. J Neurochem.

[20] Figlewicz D. P, Brot M. D, McCall A. L, Szot P (1996). Diabetes causes differential changes in CNS noradrenergic and dopaminergic neurons in the rat: a molecular study.. Brain Research.

[21] Shiota C, Woo J. T, Lindner J, Shelton K. D, Magnuson M. A (2006). Multiallelic disruption of the rictor gene in mice reveals that mTOR complex 2 is essential for fetal growth and viability.. Dev Cell.

[22] Powell S. B, Zhou X, Geyer M. A (2009). Prepulse inhibition and genetic mouse models of schizophrenia.. Behav Brain Res.

[23] Delay J, Deniker P, Harl J. M (1952). [Therapeutic use in psychiatry of phenothiazine of central elective action (4560 RP).].. Ann Med Psychol (Paris).

[24] Carlsson A, Lindqvist M (1963). Effect of chlorpromazine or haloperidol on formation of 3methoxytyramine and normetanephrine in mouse brain.. Acta Pharmacol Toxicol (Copenh).

[25] Seeman P, Lee T (1975). Antipsychotic drugs: direct correlation between clinical potency and presynaptic action on dopamine neurons.. Science.

[26] Matthysse S (1973). Antipsychotic drug actions: a clue to the neuropathology of schizophrenia?. Fed Proc.

[27] Snyder S. H (1976). The dopamine hypothesis of schizophrenia: focus on the dopamine receptor.. Am J Psychiatry.

[28] Weinberger D. R, Berman K. F, Illowsky B. P (1988). Physiological dysfunction of dorsolateral prefrontal cortex in schizophrenia. III. A new cohort and evidence for a monoaminergic mechanism.. Arch Gen Psychiatry.

[29] Howes O. D, Kapur S (2009). The dopamine hypothesis of schizophrenia: version III—the final common pathway.. Schizophr Bull.

[30] Koch M, Bubser M (1994). Deficient sensorimotor gating after 6-hydroxydopamine lesion of the rat medial prefrontal cortex is reversed by haloperidol.. Eur J Neurosci.

[31] Swerdlow N. R, Shoemaker J. M, Kuczenski R, Bongiovanni M. J, Neary A. C (2006). Forebrain D1 function and sensorimotor gating in rats: effects of D1 blockade, frontal lesions and dopamine denervation.. Neurosci Lett.

[32] Ralph R. J, Varty G. B, Kelly M. A, Wang Y. M, Caron M. G (1999). The dopamine D2, but not D3 or D4, receptor subtype is essential for the disruption of prepulse inhibition produced by amphetamine in mice.. J Neurosci.

[33] Ralph R. J, Paulus M. P, Fumagalli F, Caron M. G, Geyer M. A (2001). Prepulse inhibition deficits and perseverative motor patterns in dopamine transporter knock-out mice: differential effects of D1 and D2 receptor antagonists.. J Neurosci.

[34] Boyd F. T, Clarke D. W, Raizada M. K (1986). Insulin inhibits specific norepinephrine uptake in neuronal cultures from rat brain.. Brain Research.

[35] Figlewicz D. P, Bentson K, Ocrant I (1993). The effect of insulin on norepinephrine uptake by PC12 cells.. Brain Research Bulletin.

[36] Figlewicz D. P, Szot P, Israel P. A, Payne C, Dorsa D. M (1993). Insulin reduces norepinephrine transporter mRNA in vivo in rat locus coeruleus.. Brain Research.

[37] DeFeo-Jones D, Barnett S. F, Fu S, Hancock P. J, Haskell K. M (2005). Tumor cell sensitization to apoptotic stimuli by selective inhibition of specific Akt/PKB family members.. Mol Cancer Ther.

[38] Lindsley C. W, Zhao Z, Leister W. H, Robinson R. G, Barnett S. F (2005). Allosteric Akt (PKB) inhibitors: discovery and SAR of isozyme selective inhibitors.. Bioorg Med Chem Lett.

[39] She Q. B, Chandarlapaty S, Ye Q, Lobo J, Haskell K. M (2008). Breast tumor cells with PI3K mutation or HER2 amplification are selectively addicted to Akt signaling.. PLoS One.

[40] Kang U. G, Seo M. S, Roh M-S, Kim Y, Yoon S. C (2004). The effects of clozapine on the GSK-3-mediated signaling pathway.. FEBS Letters.

[41] Roh M. S, Seo M. S, Kim Y, Kim S. H, Jeon W. J (2007). Haloperidol and clozapine differentially regulate signals upstream of glycogen synthase kinase 3 in the rat frontal cortex.. Exp Mol Med.

[42] Yamashita M, Fukushima S, Shen H-w, Hall F. S, Uhl G. R (2006). Norepinephrine transporter blockade can normalize the prepulse inhibition deficits found in dopamine transporter knockout mice.. Neuropsychopharmacology.

[43] Kelly D. L, Buchanan R. W, Boggs D. L, McMahon R. P, Dickinson D (2009). A randomized double-blind trial of atomoxetine for cognitive impairments in 32 people with schizophrenia.. J Clin Psychiatry.

[44] Carvelli L, Moron J. A, Kahlig K. M, Ferrer J. V, Sen N (2002). PI 3-kinase regulation of dopamine uptake.. J Neurochem.

[45] Jacinto E, Facchinetti V, Liu D, Soto N, Wei S (2006). SIN1/MIP1 maintains rictor-mTOR complex integrity and regulates Akt phosphorylation and substrate specificity.. Cell.

[46] Lake C. R, Sternberg D. E, Kammen D. P. V, Ballenger J. C, Ziegler M. G (1980). Schizophrenia: elevated cerebrospinal fluid norepinephrine.. Science.

[47] Ventura R, Cabib S, Alcaro A, Orsini C, Puglisi-Allegra S (2003). Norepinephrine in the prefrontal cortex is critical for amphetamine-induced reward and mesoaccumbens dopamine release.. J Neurosci.

[48] Ryan M. C. M, Collins P, Thakore J. H (2003). Impaired fasting glucose tolerance in first-episode, drug-naive patients with schizophrenia.. Am J Psychiatry.

[49] Kanakry C. G, Li Z, Nakai Y, Sei Y, Weinberger D. R (2007). Neuregulin-1 regulates cell adhesion via an ErbB2/phosphoinositide-3 kinase/Akt-dependent pathway: potential implications for schizophrenia and cancer.. PLoS ONE.

[50] Nicodemus K. K, Marenco S, Batten A. J, Vakkalanka R, Egan M. F (2008). Serious obstetric complications interact with hypoxia-regulated/vascular-expression genes to influence schizophrenia risk.. Mol Psychiatry.

