# Supplementary figures and images for: Dysregulation of the Norepinephrine Transporter Sustains Cortical Hypodopaminergia and Schizophrenia-Like Behaviors in Neuronal Rictor Null Mice
Source: PLoS Biol. 2010 Jun 8;8(6):e1000393. doi: 10.1371/journal.pbio.1000393 (PMC2882427; doi:10.1371/journal.pbio.1000393)

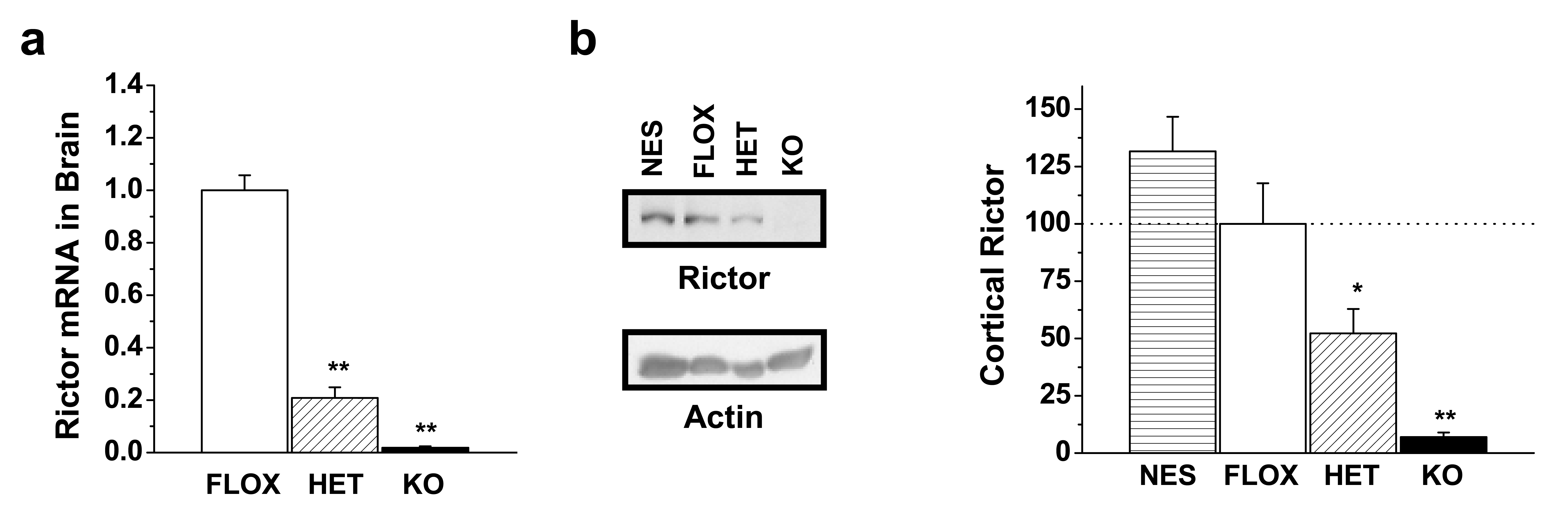

Supplement: Figure S1 — Rictor mRNA levels and protein expression in the brain are reduced in a gene-dosage dependent manner. (A) qRT-PCR confirms down-regulation of the rictor gene in HET and KO mice. Mean±s.e.m relative expression shown as a percentage of FLOX control mice; n = 4–5 animals. (B) Rictor protein levels in the cerebral cortex. Mean±s.e.m optical densities are shown as a percentage of FLOX control mice; n = 5, *p<0.05; **p<0.01 one-way ANOVA. (0.40 MB TIF) [file pbio.1000393.s001.tif]

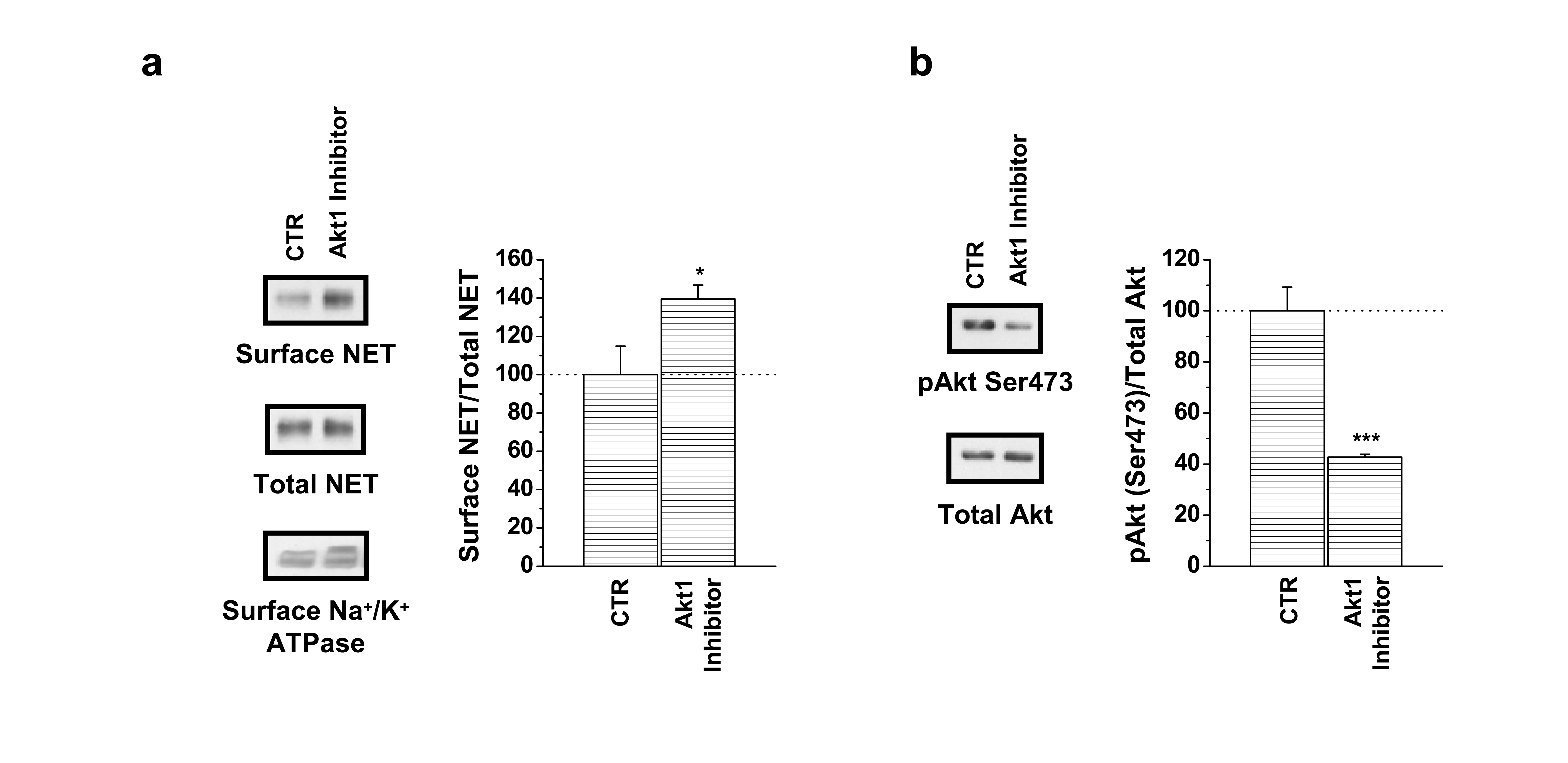

Supplement: Figure S2 — Akt1 inhibition enhances NET surface availability in cortical slices. (A) Levels of NET as measured from the different fractions (surface, total) of cortical slices from NES control mice. Slices were treated with either vehicle-DMSO (CTR) or 12 µM of the Akt1 inhibitor. Mean±s.e.m optical density were normalized to total NET and are shown as a percentage of CTR. Representative immunoblots are shown, as probed with antibodies to NET, Na+/K+ ATPase to serve as plasma membrane/loading control; n = 4, *p<0.05 Student's t test. (B) Phosphorylation of Akt on residue Ser473 measured from the same samples as (A). Mean±s.e.m of optical densities normalized to total Akt and shown as a percentage of CTR; n = 4, ***p<0.001 Student's t test. (0.67 MB TIF) [file pbio.1000393.s002.tif]
